# Using decision trees to determine participation in bundled payments in sepsis cases

**DOI:** 10.1097/MD.0000000000025902

**Published:** 2021-05-14

**Authors:** William Matzner, Deborah Freund

**Affiliations:** a Healthcare Analytics, Limited Liability Company, Simi Valley; b Claremont Graduate University, Claremont, California.

**Keywords:** cost effectiveness analysis, decision analysis, decision trees, health economics

## Abstract

**Rationale:**

The purpose of this research is to determine and develop a valid analytical method that can be easily implemented by providers to evaluate whether they should join the bundled payments for care improvement (BPCI) advanced bundled payment program, and analyze the projected impacts of BPCI advanced payment on their margins.

**Methods:**

We have developed a decision tree model that incorporates the types of sepsis encountered and the resultant typical complications and associated costs.

**Results:**

The initial cost of a sepsis episode was $30,386. Since Medicare requires that there is a 3% cost reduction under BPCI, we applied the model with a 3% cost reduction across the board. Since the model considers probabilities of the complications and readmission, there was actually a 3.36% reduction in costs when the 3% reduction was added to the model. We applied 2-way sensitivity analysis to the intensive care unit (ICU) long and short costs. We used the unbundled cost at the high end, and a 10% reduction at the low end. Per patient episode cost varied between $28,117 and $29,658. This is a 5.2% difference between low and high end. Next, we looked at varying the hospital bed (non-ICU) costs. Here the resultant cost varied between $28,708 and $29,099. This is only a 1.34% difference between low and high ends. Finally, we applied a sensitivity analysis varying the attending physician and the intensivist reimbursement fees. The result was a cost that varied between $29,191 and $29,366 which is a difference of only 0.595%.

**Conclusion:**

This is the precise environment where decision tree analysis modeling is essential. This analysis can guide the hospital in just how to allocate resources in light of the new BPCI advanced payment model.

## 1 Introduction

The cost of healthcare continues to increase with new developments in pharmacology, technology, precision medicine, higher provider fees, and a society living actively longer than ever before. This has led Center for Medicare and Medicaid Services (CMS), that runs Medicare and Medicaid as well as private insurers to develop alternate payment approaches to address the perceived weakness of the fee-for-service method. One of the new methods is bundled payments meant to bring about lower costs and higher quality by omitting fee-for-service payments which are seen as incentivizing “unnecessary” treatment without any improvement in quality.

Capitation (in which providers are given a defined sum per patient regardless of how many services are rendered in a given period of time) has been used for many years in both health maintenance organization (HMOs) and Medicare Advantage plans, also known as Medicare Part C. The Center for Medicare and Medicaid Innovation (CMMI) which is part of CMS has developed a hybrid that is in-between full capitation and fee for service called bundled payments or episode-based payments.^[1]^ In this system, reimbursements to healthcare providers (both hospitals and physicians) are made on the basis of a calculated expected cost formula for clinically defined episodes of care. As of 2012 almost one-third of medical reimbursement are now from a bundling system.^[2]^ The bane of capitation both in integrated delivery models and in HMOs has been the extent and thoroughness of patient care possible through episodes of care that involve different providers with sometimes different patient management objectives.

As a result, the purpose of this research is to determine and develop a valid analytical method that can be easily implemented by providers to evaluate whether they should join the newest bundled payment program now, bundled payments for care improvement (BPCI) advanced, and analyze the projected impacts of BPCI advanced payment on their margins. Based upon the specific inputs in the model, a hospital can determine if joining BPCI advanced is a profitable idea or not, so as to make a more informed decision when choosing whether or not to participate. Such a method should be able to consider the variables/treatments all providers who treat a specific condition use in treating patients with complex disorders.

## 2 Background

Bundling payments were first introduced by Dr Denton Cooley in 1984 at the Texas Heart Institute (THI). Dr Cooley charged a flat fee for combined hospital and physician services for coronary artery bypass grafts. THI charged an average of $13,800, when the average Medicare payment for coronary artery bypass graft (CABG) was over $24,500.^[3]^

In 2006 the Geisinger Health System tested another bundling model, also for coronary artery bypass surgery.^[3]^ This model included all preoperative, in-patient and operative care and follow up care within 90 days of the initial visit, at a fixed package price. This experiment resulted in shorter hospital stays, a 5% reduction in hospital costs, an increased chance of being discharged directly to home rather than sent to a skilled nursing facility (SNF), and a decrease in readmission rates.

## 3 Current environment

In 2012, Medicare introduced BPCI program as a result of the Affordable Care Act.^[4]^ Participants, in this voluntary program, could choose 1 of 4 payment models for 48 possible clinical episodes. Most participants opted for Model 2 which included an inpatient stay plus outpatient follow up for a period of 30, 60, or 90 days. Payments were reconciled comparing actual Medicare Payments to a payment target set by Medicare based on previous payments for similar cases. Spending generally trended lower, and in the case of hip/knee replacement there was an estimated savings of $1273 per episode, which came mostly from a reduction in SNF use in the postoperative period.

In 2016, CMMI introduced a mandatory bundled payment program for hip and knee replacement called the comprehensive joint replacement model (CJR).^[5]^ This experimental program involving 800 hospitals resulted in a savings of $1134 per episode.

In 2018, CMMI Medicare also introduced a variation called bundled payment for care improvement advanced (BPCI advanced).^[6]^ This voluntary Medicare program was designed as an alternative to the traditional fee for service payment model. In theory it was designed to support healthcare providers who invest in practice innovation and care redesign to better coordinate care and expenditures. It involves paying the physician, hospital, and other healthcare services in one single payment that is based on the expected costs during an episode of care. The incentive is for providers and suppliers to coordinate and deliver care with increased quality and less cost.

There are several differences between the original BPCI and the new BPCI advanced programs.^[7]^ First, all participants will be responsible for 90-day bundles. In the original BPCI, there was an option to choose 30, 60, or 90-day bundles. Next, there are fewer exclusions, so that the bundle includes all part A services, including the hospital stay, hospital procedures, and post-acute care services, plus all part B outpatient services unless they are not related at all to the admission diagnosis related group (DRG). Furthermore, up to 10% of payments are at risk for quality measures. There is also only a single track for treatment of outliers. And reconciliation reports will only be sent to participants bi-annually. This new iteration of BPCI is voluntary and involves a single retrospective payment for a 90-day clinical episode. This has been designed by CMS. There are 31 inpatient and 4 outpatient clinical episodes included. Payment is tied to performance on certain quality measures defined by CMS.

For BPCI Advanced, 4 payment models were available, with Model 2 being the most common.^[6]^ All payment bundles are fixed to a 90-day episode, up to 10% of payments in the bundle are at risk based upon certain quality measurements, and there is only a single track of downside financial risk, effective immediately. With downside risk, failure to improve quality or decrease costs (bills to Medicare) leads to hospitals having to return money to Medicare.

Studies of the joint replacement bundles have shown no decrease in quality and a small decrease in expenditures.^[8]^ However, there are several limitations to using joint replacement as a template for how bundling will work for different episodes. Notably, joint replacement, an elective surgery, is a standard and defined procedure. The surgery is virtually the same for each patient. Since joint replacement is an elective procedure, it can be assumed that all patients undergoing the surgery have been screened to reduce the risk of perioperative cardiovascular or pulmonary complications. In fact, studies show that most all of the cost savings for bundles of hip and knee replacement result from patients going home after being discharged for rehabilitation after discharge from the hospital or outpatient surgery center instead of to a SNF. This is not possible for many of the other medical episodes included in BPCI Advanced. For example, other episodes included in BPCI Advanced are congestive heart failure, chronic obstructive pulmonary disease, sepsis, acute myocardial infarction, and pneumonia. There is a wide variation in the degree of illness and the course of therapy with these diagnoses, and there is no uniformity in treatment guidelines for these diseases. For example, Sepsis includes 3 DRGs which range from uncomplicated sepsis to septic shock. Unlike elective surgery such as hip and knee replacements, patients cannot be screened to avoid complications. In fact, many sepsis patients develop complications and/or have significant comorbidities resulting in extreme variation in length of stay (LOS) and unpredictable costs associated with each hospitalization. Most cases would likely not to need to go to SNF after discharge from a hospital but would most likely need home health care and close medical follow up to avoid readmission.^[9]^

Within the joint replacement modeling, the decision of whether a provider should participate is generally simple to calculate. However, in cases where there is a wide variation in cost factors, a model able to address numerous variables attributable to different patients is imperative for a provider to make any informed decision about whether to participate in BPCI Advanced. Our tested and recommended model for these calculations is a Decision Tree Model. Decision tree models offer both the flexibility and complexity of interaction to more accurately predict costs than just a linear model which is commonly used. Since sepsis is a complicated disease that can lead to many possible outcomes that would affect costs, this type of modeling lends itself to such an analysis.

Previous studies of cost analysis on BPCI have only compared total costs to what has been predicted. Meyer^[10]^ found that bundled payments cut spending on joint replacement but not for other conditions. Agarwal et al^[11]^ looked at the impact of bundled payment on healthcare spending utilization, and quality and came to a similar conclusion. The Rand Corporation^[12]^ found costs went down only 5% compared with 15% predicted. All such studies look at total cost but did not incorporate different clinical outcomes.

We have developed a decision tree model that incorporates the various types of sepsis that are encountered and the resultant typical complications and their associated costs. Sepsis affects 1.7 million adults in the United States each year and potentially contributes to 250,000 deaths. It is present in 34% to 53% of hospitalizations in which the patients died.^[13]^ Sepsis is an overwhelming bacterial infection in the body. Bacteria are present within the bloodstream and can lead to organ damage, especially to the kidneys and lungs, and the vascular system. This can become septic shock, which has risen by 10% in the last 3 years. These patients are intubated in the intensive care unit due to respiratory failure, are on dialysis for acute renal failure, and on vasopressor medication to keep their blood pressures high enough to perfuse their brains. Only with the aggressive use of IV antibiotics, IV fluids, and other supportive care will the patient even survive. Reported mortality rates vary between 37% and 45%. Hospital charges are now up from $58,000 to $70,000 per case (although actual costs are less).^[14]^ Overall, hospitals spent $1.5 billion more in 2018 on sepsis than in 2015.^[15]^

## 4 Methods

**Figure 1:**
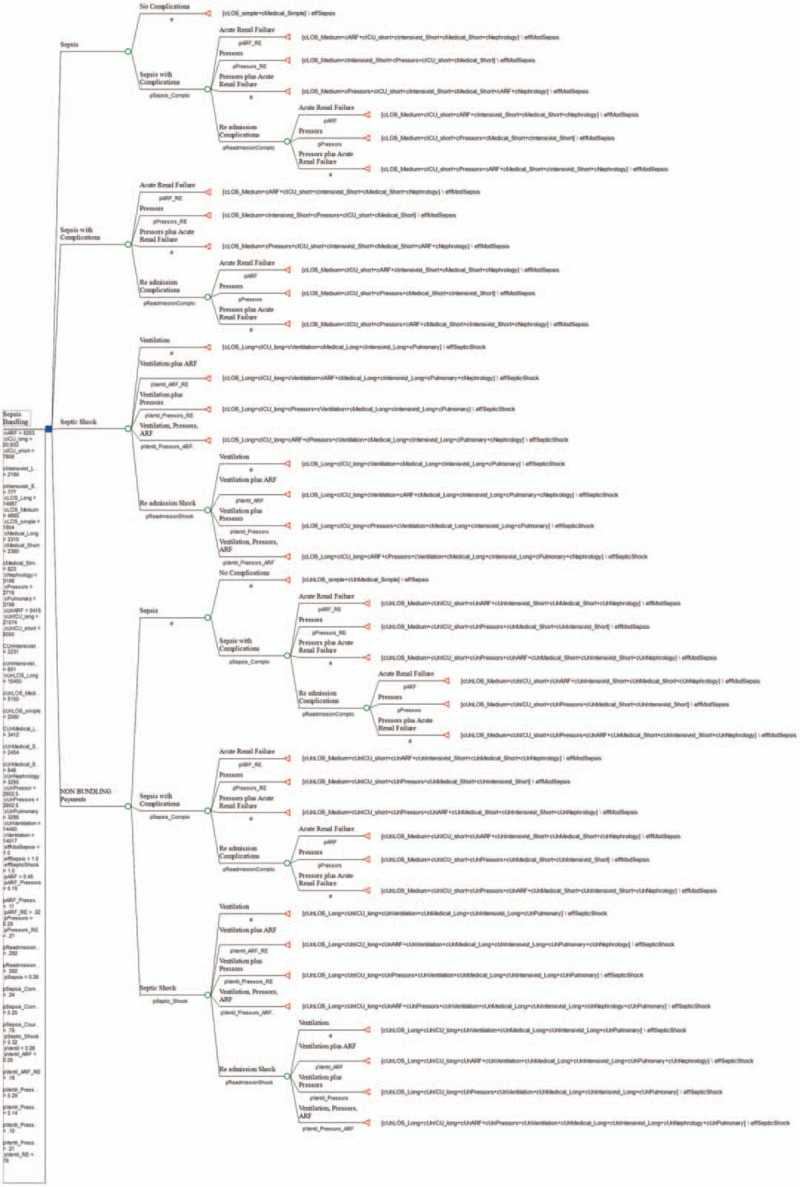
Decision tree model. The decision tree illustrates with this range of variables utilized that bundling is less expensive, at the rate of 3%.

The decision tree model was developed in Tree Age Pro Version 19.2.1. We constructed 2 branches, 1 for bundled payments, and 1 for non-bundled payments. Figure 1 shows the entire decision tree.

There was no need for an ethics committee as we used no individual patient data for the research and did not offer any type of treatment in this study.

The possible complications which are analyzed in the model include: acute renal failure, respiratory failure, hypotension, and readmission for sepsis within 1 month of the first admission. However, based on past experience, on a provider-by-provider basis, other complications can be included. Both the number of complications and the specific complications are virtually unlimited and may be customized to reflect the actual use of an individual provider organization or groups of organizations.

In this decision tree model there are different “branches” for the different treatment variations, and each branch has an associated cost and probability of occurrence. In the case of the sepsis model for BPCI, there are 2 main branches, 1 for the bundled payment plan (BPCI) and 1 for the unbundled, traditional fee for service payment plan. The branches are identical except for the costs of the different branches. Sepsis has 3 major branches: systemic inflammatory response syndrome (SIRS), sepsis (with complications but not septic shock), and septic shock. The major complications include acute renal failure, hypotension (low blood pressure) that needs pharmacologic support by medications called vasoconstrictors, and septic shock where the patient is in respiratory failure and is intubated. Costs for these complications include the cost of the ICU, the ventilator, dialysis for renal failure, and cost and administration of the vasoconstrictors. These costs were derived from the medical literature and are the average national costs of ventilator, and dialysis management.^[16,17]^ The cost of vasoconstrictors came from the pharmaceutical company that manufactures it.^[18]^ Furthermore, the cost per day of the ICU and subsequent regular floor beds are added to the cost. We got these costs from the CFOs of community/hospitals in the California Hospital Association. These costs therefore are illustrative how the model works, but as costs for ICU and regular floor beds may vary across the country, one may not be able to necessarily rely on the specific conclusions of this manuscript. However, if a hospital includes their own values, the model will give an accurate representation of the expected values for their facility. This cost was the per day cost to stay in either an ICU bed or a regular floor bed. We used the average length of stay from the literature in the ICU and subsequent regular floor bed for septic shock, sepsis with complications, and simple sepsis. Finally, we added the daily cost of the physicians involved in the care of each patient. We assumed that the hospital will reimburse the physician at the Medicare payment rate. This rate was derived from the Medicare reimbursement for physicians for their particular level of service. One of the branches of the model is devoted to readmission within the 90-day period prescribed by Medicare BPCI. Therefore, there are 4 branches for the main possible outcomes: SIRS, sepsis (with complications), septic shock, and readmission for sepsis. For both bundled and unbundled, the probabilities are identical—it is only the costs that vary.

The probability of SIRS is 26%, sepsis (with complications) is 24%, septic shock is 32%, and the probability of readmission is 29.2%.^[19]^ Essentially, there is an expected value calculated (probability times the cost) for each branch, and the expected values of costs are then summed to determine the cost of each branch. The cost of a sepsis admission is the sum of the expected values of all the branches for a particular arm of the model.

Data on hospital costs (cost per day in ICU or regular floor bed) were given to us by hospitals that are members of the California Hospital Association. While these data are not representative they are not meant to be. We use them only to illustrate the methods of using decision trees. A similar analysis can be conducted for private patients as well. The payment of physicians was derived from the Medicare Fee Schedule for physicians for the appropriate current procedural termino (CPT) codes. Costs of dialysis and ventilation were derived from the literature. Costs of vasopressors were taken from the drug manufacturer information on their website.

The Payoffs for the decision tree, including all costs and probabilities, are listed in Table 1.

**Table 1:** Payoffs.

Name	Description	Root definition
cARF	Cost of acute renal failure	5253
cICU_long	Cost of long ICU stay	20,933
cICU_short	Cost of short LOS in ICU	7809
cIntensivist_Long	Cost of intensivist in ICU long stay	2164
cIntensivist_Short	Cost of intensivist in ICU short stay	777
cLOS_Long	Cost of LOS long	14,987
cLOS_Medium	Cost of LOS for medium case	4995
cLOS_simple	Cost of LOS simple sepsis	1654
cMedical_Long	Cost of medical doctor long stay	3310
cMedical_Short	Cost of medical doctor short stay	2380
cMedical_Simple	Cost of medical doctor SIRS	823
cNephrology	Cost of nephrologist	3196
cPressors	Cost of pressors	2718
cPulmonary	Cost of pulmonologist	3196
cUICU_short	Cost of short LOS in ICU-unbundled	8043
cUnARF	Cost of acute renal failure unbundled	5415
cUnICU_long	Cost of unbundled ICU long	21,574
cUnICU_short	Cost of unbundled ICU short	8050
CUnIntensivist_Long	Cost of intensivist in ICU long stay unbundled	2231
cUnIntensivist_Short	Cost of intensivist in ICU short stay unbundled	801
cUnLOS_Long	Cost of unbundled LOS long	15,450
cUnLOS_Medium	Cost of unbundled LOS medium	5150
cUnLOS_simple	Cost of unbundled LOS simple	2060
CUnMedical_Long	Cost of medical doctor long stay unbundled	3412
cUnMedical_Short	Cost of medical doctor short stay unbundled	2454
cUnMedical_Simple	Cost of medical doctor SIRS unbundled	848
cUnNephrology	Cost of nephrologist unbundled	3295
cUnPressor	Cost of pressors unbundled	2802.5
cUnPressors	Cost of pressors unbundled	2802.5
cUnPulmonary	Cost of pulmonologist unbundled	3295
cUnVentilation	Cost of ventilating patient unbundled	14,450
cVentilation	Cost of ventilating patient	14,017
effModSepsis	Effectiveness of septic shock	1.0
effSepsis	Effectiveness of simple sepsis	1.0
effSepticShock	Effectiveness of septic shock	1.0
pARF	Probability of ARF	0.45
pARF_Pressors	Probability of ARF and pressors during hospital	0.15
pARF_Pressors_RE	Probability of ARF and pressors during hospital-readmit	0.11
pARF_RE	Probability of acute renal failure readmit	0.32
pPressors	Probability of use of pressors in complicated sepsis	0.29
pPressors_RE	Probability of use of pressors in complicated sepsis-readmit	0.21
pReadmissionComplic	Probability of readmission complication	0.292
pReadmissionShock	Probability of readmission septic shock	0.292
pSepsis	Probability of SIRS	0.26
pSepsis_Comp_Course	Probability that SIRS becomes complicated	0.24
pSepsis_Complic	Probability of complicated sepsis	0.25
pSepsis_Course	Probability of SIRS	0.75
pSeptic_Shock	Probability of septic shock	0.32
pVentil	Probability of ventilation alone septic shock	0.26
pVentil_ARF	Probability of ventilator and ARF in septic shock	0.25
pVentil_ARF_RE	Probability of ventilator plus acute renal failure	0.18
pVentil_Pressors	Probability ventilation and pressors in septic shock	0.29
pVentil_Pressors_ARF	Probability of pressors and ARF and ventilation in septic shock	0.14
pVentil_Pressors_ARF_RE	Probability of pressors and ventilator and ARF in septic shock-readmit	0.10
pVentil_Pressors_RE	Probability of ventilator and pressors in septic shock-readmit	0.21
pVentil_RE	Probability of ventilation alone septic shock-readmit	0.18

For this study, the expected value (EV) of each branch (probability times cost) was calculated, then the EV of all branches summed to get total cost. Two-way sensitivity analysis was also calculated using the software to explore the effects of changing various costs upon the overall model.

## 5 Results

We used the model to realistically analyze the effects of varying certain costs on the overall cost to the hospital of a sepsis admission. In that way, hospitals can determine whether likely revenue from bundled payments will be large enough to allow them to both provide care more efficiently and also take on the risk and be rewarded by a share in the savings. The decision tree takes into account 3 major complications: acute renal failure, hypotension, and septic shock with respiratory failure. It also considers the 30-day readmission rate for sepsis, which is quite high. Costs included in the model are short (3.5 days) and long (9.5 days) ICU stay, short (5.1 days) and long (15.4 days) length of stay in a regular room, dialysis costs, respirator costs, medication costs (specifically vasopressors), and costs of the attending physician, intensivist, pulmonologist, and nephrologist.

The results are summarized in Table 2. The initial cost of a sepsis episode was $30,386. Since Medicare requires that there is a 3% cost reduction under BPCI, we applied the model with a 3% cost reduction across the board. Since the model considers probabilities of the complications and readmission, there was actually a 3.36% reduction in costs when the 3% reduction was added to the model.

**Table 2:** Illustrates both the ranges between high and low costs aggregated from among participating hospitals, but also the percent differential.

Parameter	Low	High	PCT Diff
Baseline	$ 29,366	$ 30,386	3.36
ICU	$ 28,117	$ 29,658	5.2
Hosp Bed	$ 28,708	$ 29,099	1.34
Physician	$ 29,191	$ 29,366	0.595

As is shown, it is the ICU cost that carries the greatest variances and therefore the greatest opportunity for cost management. The least volatile is the physician charge. Low: 10% below bundled cost. High: Unbundled cost. Bundled = 97% unbundled (BPCI requiring 3% cost savings). ICU = ICU long and short stay. Hosp Bed = regular bed LOS cost. Physician = cost of internist and hospitalist.

We next applied 2-way sensitivity analysis to the model and monitored how this affected the model. The purpose of this analysis is to evaluate how either a change in therapy, or a change in costs administratively, can optimize the revenue hospitals and physicians receive under the bundled payment system. Since total revenue is fixed under bundled payments, it is important to minimize costs either by changes in the therapeutic regimen, or a change in costs for a particular episode of care.

When deciding what may need to change, it is imperative that one knows what would have the largest impact for a particular change in therapy or cost. This is where sensitivity analysis in a cost effectiveness model can provide insight into what aspect of the care episode needs to be examined more closely.

First, we applied the analysis to the ICU long and short costs. We used the unbundled cost at the high end, and a 10% reduction at the low end. The result was that the per patient episode cost varied between $28,117 and $29,658. This is a 5.2% difference between low and high end. Next, we looked at varying the hospital bed (non-ICU) costs. Again, we used the unbundled cost at the high end and a 10% reduction at the low end. Here the resultant cost varied between $28,708 and $29,099. This is only a 1.34% difference between the low and high ends. Finally, we applied a sensitivity analysis varying the attending physician and the intensivist reimbursement fees. The result was a resultant cost that varied between $29,191 and $29,366 which is a difference of only 0.595%.

## 6 How to use this analysis

This analysis can guide the hospital in just how to allocate resources in light of the new BPCI advanced payment model. Since revenue is essentially fixed and predetermined, it is imperative that the hospital analyze and then cut back unnecessary costs while still maintaining excellent delivery of healthcare to the sepsis patient. Our analysis shows that the biggest impact would be to cut back on the length of ICU care, and/or to cut some of the costs that are incurred in the ICU.

One can see that a combination of savings through medical methodology plus administrative efficiency can lead to a savings of $3873 per admission. See Table 3. Since revenue is fixed, this would go straight to profit. If using the same example of a hospital with 25 ICU beds, based on the available data it would save $2,649,132 during the course of year which would go to profit under a fixed revenue model such as BPCI advanced.

**Table 3:** Illustrates the practical application of the modeling to project cost savings in the ICE, the major charge/cost component, when adjusted for individual hospitals.

No. of ICU beds	ICU bed days	% Sepsis	Sepsis Bd/d	Avg LOS	Calculated admissions	Savings per admission	Total annual savings
25	9125	45%	4106	6.0	684	$ 3873	$ 2,649,132

Calculations for Table 3: Enter actual number of ICU Beds, Bed Day calculated as ICU beds times 365, percent sepsis based on reference data averages, Sepsis bed days calculated as percent sepsis times ICU bed days. Calculated sepsis admissions, based on sepsis bed days divided by average sepsis LOS, from reference material, savings per admission is calculated sepsis admissions times the difference between Low and High ICU costs from Table 2 above. Total annual savings is the savings per admission times the calculated sepsis admissions. ICU = intensive care unit, LOS = length of stay.

## 7 Discussion

Bundling payments versus the traditional fee-for-service (FFS) presents a different paradigm in not only how to treat the patients and communicate with specialists, but also how hospitals and hospital administrators and physicians can undertake a different approach to revenue generation in light of the costs that are experienced in a typical episode of care. In the previous payment methods, it is simple to charge for certain fees as it is just a matter of submitting a number where the payment exceeds the costs so a profit can be obtained. In traditional existing payment methods, charges are effectively agreed to base upon existing Medicare and Medicaid Payments or in the case of Private Insurers contractual terms negotiated between insurers and the physician, hospital provider or system and calculated through the coding schemes that apply to the different providers. DRG groupers and other software applications attempt to maximize those charges, but those are based on small changes with the system rather than changes in physician care to reduce costs.

This is not the case with the bundled payment models. The interactions among hospital, primary physician, and all the physician specialists are more complex. They must discuss new styles of practice and where patient care costs may be reduced without jeopardizing patient outcomes. Furthermore, payment is somewhat based on outcome which is not the case at all in a FFS model. The combined interrelatedness and matrix interactions require a much more complex analysis in order to decide if the hospital is making money or losing money for a particular disease/diagnosis.

This is the precise environment where decision tree analysis modeling is essential. The probabilities of specific outcomes were obtained from the literature. Decision tree modeling takes into account the assorted itemized costs associated with a readmission, which is especially important in a bundled payment model, as readmissions in 90 days will not be paid additional revenues. Even without different therapeutic arms or different measurements of quality adjusted light years (as in a cost effectiveness analysis), this analysis can give much insight into what costs to look at while operating a sepsis case.

Sensitivity analysis of the model provides insight into how a hospital can identify specific cost centers for management or process changes to affect costs and improve margins within the framework of a bundled payment. We were able to show that affecting the costs of the ICU stay had the most impact on overall costs whereas changing the payments to the physicians registered minimal changes to costs at all. This type of analysis can therefore direct the administrators to concentrate on affecting costs to the particular areas that have the most financial impact.

There are however some limitations to this analysis. Firstly, many cases of sepsis can deteriorate from, for example, SIRS to sepsis with complications, or even to septic shock. This is difficult to model and was one thing we did not incorporate. Perhaps one can add the number of cases converted from SIRS to sepsis with complications, and call that the final probability of sepsis with complications. Notable that the complication rate would likely be minimized as practicable and not primarily related to financial compensation as the reputation of the hospital and its physicians in treating sepsis is logically the most important driver. The numbers used in the analysis are examples, and in practice one must use numbers generated from the local hospital to make up for geographical differences and differences in the success of how patients with sepsis are treated in a particular hospital. So even though the numbers presented in this paper are examples, in practice one can use the actual local numbers to help in making a decision about whether or not a hospital wants to participate in BPCI advanced (currently voluntary).

Sepsis, defined as infection with associated organ failure, was identified during the ICU stay in 2973 (29.5%) patients, including in 1808 (18.0%) already at ICU admission as of 2006.^[20]^

Occurrence rates of sepsis varied from 13.6% to 39.3% in the different regions. Patients with sepsis accounted for 45% of ICU bed days and 33% of hospital bed days. The ICU length of stay (LOS) was between 4 and 8 days and the median hospital LOS was 18 days.^[21]^ If a hospital has 25 ICU beds, which accounts for 9125 ICU bed days, then at a savings of $3873 per admission based on cost containment in the ICU, a hospital would wind up with an additional $2,649,132 in profit if done correctly. (Table 3)

## 8 Appendix-example

To demonstrate how the model works, a 60-year-old woman, who is diabetic, comes to the emergency room. She complains of exhaustion and has difficulty breathing. On presentation, she is hypotensive (systolic blood pressure of 70), and also is in acute renal failure (BUN 80, Cr 5.7). Her respirations are labored and as a result, she is intubated. Her urine shows many bacteria and TNTC WBC. Her blood sugar is 480.

She is immediately started on fluid resuscitation (normal saline 1-L bolus followed by 120 cm^3^/h), and then she is started on a neosynephrine drip to maintain her blood pressure. After blood and urine cultures are obtained, she is given IV Rocephin and IV Imipenem antibiotics. She is also started on an insulin drip.^[22,23]^

The patient is transferred to the ICU, where she remains for 5 days. During this time she has to acute hemodialysis 4 times, and it takes 3 days to reduce the neosynephrine so that she maintains a systolic BP of 95 on her own. By the 5th day, the pulmonologist was able to remove her from the ventilator and she was extubated. After urine and blood cultures came back positive for a resistive form of *Escherichia coli*, she is maintained on the IV Imipenem to which it was sensitive.

The patient spends the next 6 days in a ward bed. She continues on IV antibiotics and IV fluids until discharge, but her renal function improved (BUN 32, Cr. 1.2) so that she does not need hemodialysis any longer. Her breathing is adequate and she oxygenated well. Her blood sugars are controlled with oral agents and subcutaneous insulin. She was discharged home in good condition.

Now unlike the decision tree, in this example the probability of certain things happening is one (and the other branches 0) so the costs are just summed. The costs for this stay: 

**Table:** 

ICU	11,500
...Floor stay	6000
Dialysis	680
Ventilator	2610
Pressor	885
Nephro	3295
Pulmon	3295
Intensivist	1192
Primary Care	2231
Total	31,688

In the model, there is a probability that each branch will occur. We multiply the probability of each occurrence (here the probability of septic shock is 14%) times the cost, so in the overall analysis this example would contribute (0.14 × $31,688) or $4436 towards the overall costs. When one does this with the costs of the different scenarios and sums the EVs, the calculated cost is $30,386, which is reported in the results section.

## Author contributions

**Conceptualization:** William Matzner, Deborah Freund.

**Data curation:** William Matzner.

**Formal analysis:** William Matzner, Deborah Freund.

**Methodology:** Deborah Freund, William Matzner.

**Writing – original draft:** William Matzner.

**Writing – review & editing:** Deborah Freund.
